# Disordered RuO_2_ exhibits two dimensional, low-mobility transport and a metal–insulator transition

**DOI:** 10.1038/srep21836

**Published:** 2016-02-26

**Authors:** M. S. Osofsky, C. M. Krowne, K. M. Charipar, K. Bussmann, C. N. Chervin, I. R. Pala, D. R. Rolison

**Affiliations:** 1 Materials and Sensors Branch (Code 6360), U.S. Naval Research Laboratory, Washington, DC 20375, USA; 2 Electromagnetics Technology Branch (Code 6850), U.S. Naval Research Laboratory, Washington, DC 20375, USA; 3 Surface Chemistry Branch (Code 6170), U.S. Naval Research Laboratory, Washington, DC 20375, USA

## Abstract

The discovery of low-dimensional metallic systems such as high-mobility metal oxide field-effect transistors, the cuprate superconductors, and conducting oxide interfaces (*e.g.*, LaAlO_3_/SrTiO_3_) has stimulated research into the nature of electronic transport in two-dimensional systems given that the seminal theory for transport in disordered metals predicts that the metallic state cannot exist in two dimensions (2D). In this report, we demonstrate the existence of a metal–insulator transition (MIT) in highly disordered RuO_2_ nanoskins with carrier concentrations that are one-to-six orders of magnitude higher and with mobilities that are one-to-six orders of magnitude lower than those reported previously for 2D oxides. The presence of an MIT and the accompanying atypical electronic characteristics place this form of the oxide in a highly diffusive, strong disorder regime and establishes the existence of a metallic state in 2D that is analogous to the three-dimensional case.

The existence of metallic behavior reported for several 2D materials violates the famous prediction of Abrahams, Anderson, Licciardello, and Ramakrishnan^1^ that all 2D systems must be localized regardless of the degree of disorder. The discovery of a metallic state in high-mobility metal oxide field-effect transistors (HMFET)^2^,^3^,^4^,^5^,^6^,^7^ motivated several theoretical approaches that included electron–electron interactions to screen disorder. These models adequately described the results for low carrier concentration, high-mobility systems^8^,^9^, but are not applicable to the case of highly disordered 2D metals. The HMFET results also motivated the development of a general scaling model for the 2D MIT that may also be applicable to highly disordered systems^10^. The presence of a metallic state in highly disordered 2D systems with very low mobility, the situation addressed by Abrahams *et al.*^1^ indicates that either a modification of the existing theory^10^ or a new theoretical approach for the 2D MIT is needed.

In conventional metals with low disorder, the metallic state is characterized by decreasing resistivity with decreasing temperature as described by the well-known Boltzmann or Bloch–Gruneisen transport theories. These theories are predicated on the existence of plane wave electrons with long mean free paths. Because disorder severely reduces the mean free path and the character of metallic transport changes, those models must be replaced with a quantum diffusion description. For highly disordered conductors in which resistivity does not decrease with decreasing temperature, a more fundamental definition of metallic transport is needed in which diffusive electrons extend throughout the material at T = 0. The disorder-driven MIT is then described as a quantum phase transition characterized by extended states for the metallic phase and by localized states for the insulating phase^11^,^12^. In three dimensions, the phase diagram has four regions^13^: insulating, critical, amorphous metal, and conventional metal (Fig. 1) with the observed properties determined by the position of the Fermi energy. It should be noted that this model is not rigorously correct due to the use of the density of states in the conductance in scaling relations for the renormalization group. However, it has proven to be a useful model for analyzing experimental data (see references 11, 14, and 15). In the conventional metal region, transport is controlled by electron–phonon scattering as described in the usual manner by Boltzmann transport with conductivity increasing with decreasing temperature. In the amorphous metal region, transport is controlled by weak localization with enhanced electron–electron interactions where conductivity follows the square root of temperature (σ = σ_0_ + σ_1_T^½^)^11^,^13^,^14^,^15^,^16^. Here, the electronic conductivity drops with decreasing temperature, but extrapolates to a finite value at T = 0.

The 2D case is quite different. The original theoretical work that described the 3D MIT^1^ also predicted that all 2D systems will be insulators with σ ~ log(T). Later work indicated that this log(T) behavior would also be a consequence of enhanced electron–electron interactions in a diffusive 2D system^17^. Indeed, conductivity that followed log(T) behavior was observed for Si MOSFETs^18^,^19^ and ultrathin films^20^,^21^,^22^,^23^,^24^ (~10-nm thick). A drawback arises when using either the thin film or transistor-based systems to explore the match of experimental results to log(T) behavior because the effective thickness of the conducting layer varies either due to changing film thickness or the spatial extent of the gated charge layer in the FET. Later work on Si HMFETs and other systems^2^,^3^,^4^,^5^,^6^,^7^ indicated that an MIT was actually possible in 2D systems. Some of these results were modeled using a renormalization group theory of electron–electron interactions where high-mobility carriers present at low carrier concentration screen the lattice disorder^8^. A reexamination of the original scaling arguments of reference 1 concluded that a 2D MIT was indeed possible for any level of disorder^10^.

## Preparing disordered RuO_2_ and tuning the transport properties

To study the highly disordered case, we prepared disordered 10-, 20-, and 30-nm thick films of ruthenium dioxide, RuO_2_, which in its anhydrous rutile crystalline form is a high carrier concentration metallic oxide (n ~ 10^23^ cm^−3^). These film-thickness values (verified by atomic force microscopy) are consistent with systems that exhibited 2D behavior such as the 40-nm–thick disordered Si_1−x_Au_x_ films^16^ and interfacial oxides^25^. The high cost of ruthenium has motivated our development of a liquid-phase, subambient temperature technique to synthesize ultrathin films of ruthenium dioxide^26^,^27^ for applications in which the properties are surface-dominated such as charge storage for pulse power^28^ and electrocatalysis^26^,^29^,^30^. On planar substrates, ~10 nm of oxide is deposited (designated RuO_2_ nanoskin); repeating the solution-phase deposition adds an additional 10-nm of oxide per cycle. Previous work demonstrated that the close-packed nanoparticulate morphology of the RuO_2_ nanoskin is unchanged with calcination up to at least 200 °C in air or argon. The X-ray photoelectron (XPS) and diffraction (XRD) results from the same study show that the chemical state and atomic structure of the RuO_2_ remains invariant up to 200 °C^27^. After deposition, the films were patterned using a 266-nm diode-pumped solid-state laser into a configuration that enabled standard four-probe resistivity and Hall measurements (Fig. 2(a) inset). Hall measurements were made in a Quantum Design Physical Property Measurement System (PPMS) between 1.75 K and 305 K for –8T ≤ B ≤ 8T.

To access higher conductivity regions of the metallic rutile phase and serve as a crystalline control for the solution-deposited ruthenia, 10- 20- and 30-nm–thick RuO_2_ films were deposited onto SiN/Si substrates held at 600 °C by reactive sputtering from a Ru metal target in a 2:1 ratio of Ar:O_2_ at a pressure of 3 mTorr. The XRD analysis of the physically sputtered films showed crystalline rutile RuO_2_ and no other competing phases. These films, denoted as “sputter 1” and “sputter 2”, exhibit conventional metallic behavior with resistance decreasing with decreasing temperature (inset, Fig. 3).

As grown on 3D-structured substrates and on planar, smooth substrates the solution-deposited films consist of uniform, high-impedance, X-ray amorphous 2–3-nm grains^26^,^27^. By calcining at moderate temperatures (~100–200 °C), the RuO_2_ nanoskins retain their X-ray amorphous structure (see supplemental information), yet exhibit a decrease in resistivity to that characteristic of a disordered metal, less than 1 mΩ−cm. The equivalent 2D resistance is almost two orders of magnitude lower than the quantum resistance per square, h/2e^2^, at room temperature (Fig. 2a). The RuO_2_ nanoskins can thus be driven through the MIT by systematically calcining at increasing temperatures *without changing the thickness* (Fig. 3). This shape invariance makes this system ideal for studies of the two-dimensional MIT.

## 2D vs. 3D transport

The temperature-dependent electronic transport data were plotted as conductivity *vs*. T^1/2^, the expected relationship for 3D behavior, because conductivity is the relevant quantity for determining whether a material is a metal or an insulator. The 3D behavior was considered first because a 2D MIT was not anticipated for these ultrathin films. While there is a clear increase in the overall values of the conductivity with calcination temperature and a clear transition from insulating to metallic states similar to that seen in disordered FETs^31^, the curves clearly do not fit the 3D temperature dependence of T^½^ (Fig. 4).

When plotting the conductance data as a function of log(T), the behavior expected for homogeneously disordered 2D systems^11^ and granular systems^32^, yields a better fit to the data (Fig. 5). Because the RuO_2_ nanoskins consist of amorphous grains as determined by electron diffraction^27^ and grazing-incidence XRD (supplemental information) rather than metallic particles embedded in an insulating matrix, it is apparent that they must be treated as a uniform disordered system rather than as a granular metal. This structural assignment is confirmed by the continuous increase in carrier concentration with calcination (Fig. 2b). The presence of granular behavior can also be ruled out because granular systems exhibit σ ~ log(T) at high temperatures and transport properties characteristic of disordered metals, *i.e.*, weak-localization and enhanced electron-electron interactions, at low temperatures^32^. It is clear from Fig. 5 that the log(T) behavior only manifests at low temperatures.

Several features are evident from these plots. For RuO_2_ nanoskins that were calcined at the highest temperatures, the conductance is metallic with flat temperature dependences at low temperatures. For lower-temperature calcinations (between 180 °C and 190 °C for the 20-nm–thick nanoskins and between 160 °C and 165 °C for the 30-nm–thick nanoskins; supplemental material) there is a transition from the metallic to a weakly localized insulator phase with σ ~ log(T). This type of “transition,” where the slope of the temperature dependence changes, is often identified as the MIT and was predicted to occur for ρ ~ 200 μΩ-cm in 3D^13^. Finally, the temperature dependence changes to a more severe localized behavior for the lowest calcination temperatures (between 160 °C and 165 °C for the 20-nm–thick nanoskins and between 140 °C and 145 °C for the 30-nm–thick nanoskins). This change in transport behavior signifies the transition in these ultrathin films from weakly localized carriers to strongly localized insulators^19^.

These curves clearly show that the RuO_2_ nanoskins exhibit 2D transport characteristics at low temperatures. The relevant low-temperature data were fitted to


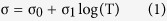


a generic function that describes 2D conductivity in disordered metals^11^. The fits are shown as solid lines in Fig. 5. Estimates of the phase coherence length for the metallic samples indicate that films can be treated as 2D systems (supplemental Information).

One of the key issues in understanding the MIT is the slope of the so-called “mobility edge,” the critical phase line that describes the scaling of the MIT. Early work by Mott^33^ on 3D systems suggested that this line is discontinuous and that an abrupt transition occurs from the metal to insulating phases (hence the term “edge”). Later work showed that this transition is continuous. In three dimensions, this line is usually defined as the relationship between a driving parameter, generically labeled as p, and 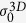, the value of conductivity extrapolated to T = 0^11^,^12^,^13^,^14^,^15^,^16^. The usual formulation is 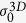 ~ (p − p_c_)^ν^ where p_c_ is the critical value of p (*i.e*., where 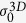 = 0) and where ν is a critical exponent^1^,^11^,^12^,^13^,^14^,^15^,^16^,^34^. Experimentally, p is often the carrier concentration. Another choice for p is the bare conductivity that can be approximated by the room-temperature conductivity^14^. It has been shown that in three dimensions, ν = ½ in Si:P^35^, while ν = 1 in disordered metals^11^,^13^,^14^,^15^,^16^.

**Figure 1:**
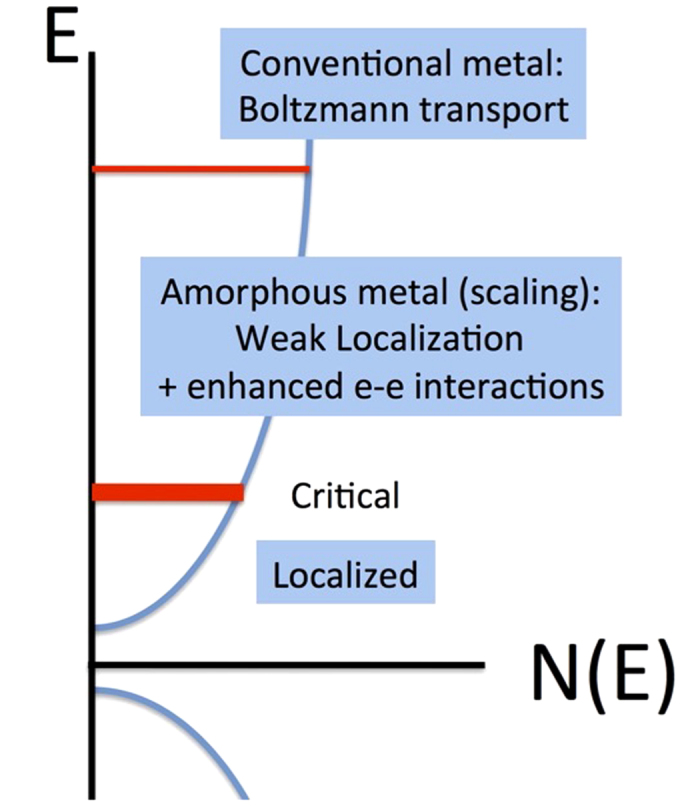
Schematic of the generic phase diagram for the metal–insulator transition. The continuous transition from amorphous to conventional metal phases is represented by a line in this rendering.

In two dimensions, the analogue to the “amorphous metal” phase shown in Fig. 1 is an atypical insulator phase and this analysis is complicated by the fact that the data cannot be extrapolated to T = 0. In this case, one can replace σ_0_ from equation (1) with σ_1K_ so that 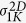 ~ (σ_300Κ_ − σ_c_)^ν^ where σ_c_ is the value of σ_300K_ for which σ_1K_ = 0. The mobility edges for the three thicknesses of solution-deposited RuO_2_ are plotted in Fig. 6. These plots clearly show that the transition is continuous with ν = 1, similar to many disordered 3D systems.

## High carrier concentration and low mobility

**Figure 2:**
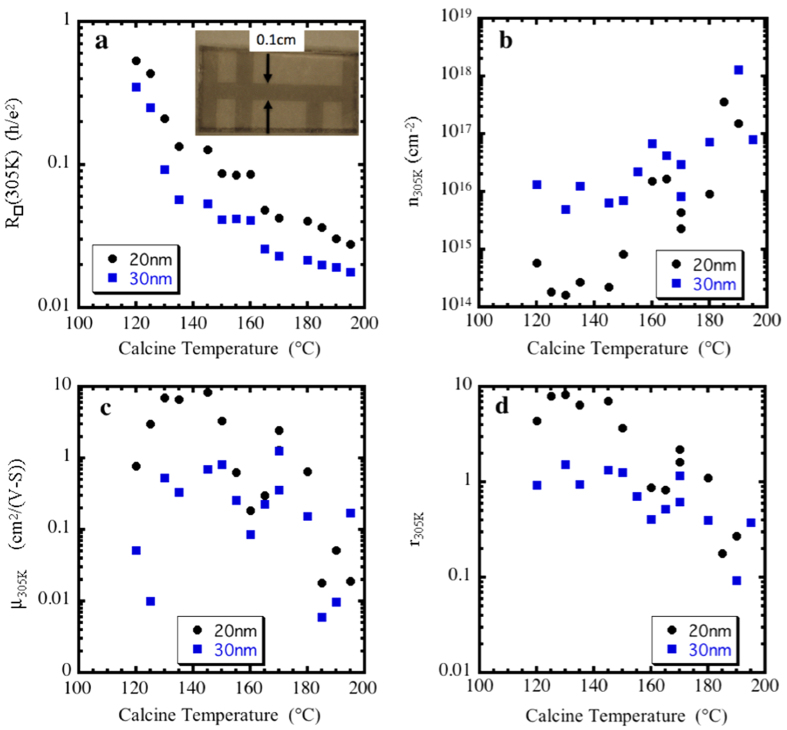
Room-temperature transport properties of RuO_2_ nanosheets. (**a**) R/◻ (plotted in units of quantum resistance, h/e^2^); (**b**) carrier concentration; (**c**) mobility; and (**d**) the “r_s_” parameter (labeled r_305K_) as a function of calcination temperature for the 20-nm (on SiO_2_ substrate) and 30-nm (on Al_2_O_3_ substrate) thick RuO_2_ nanosheets. Inset (**a**) geometry of the laser-patterned films.

**Figure 3:**
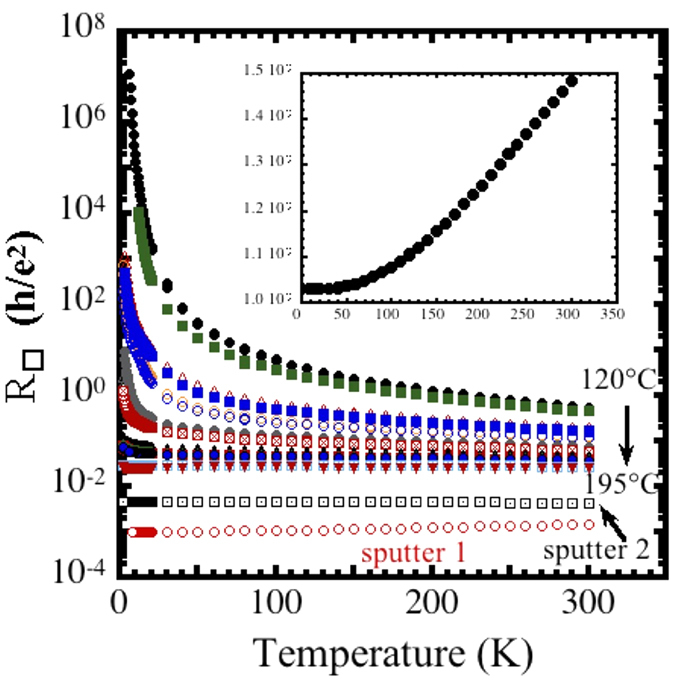
Sheet resistance as a function of temperature of thin-film disordered and crystalline RuO_2_. R/◻ (plotted in units of quantum resistance, h/e^2^) obtained from 1.75 to305 K for 20-nm solution-deposited RuO_2_ nanosheet as a function of calcination temperature. Inset: blow-up of the data for a 20-nm sputtered RuO_2_ film on a SiN/Si substrate grown at 600 °C (sputter 1) that exhibits conventional metallic behavior with resistance decreasing with decreasing temperature.

**Figure 4:**
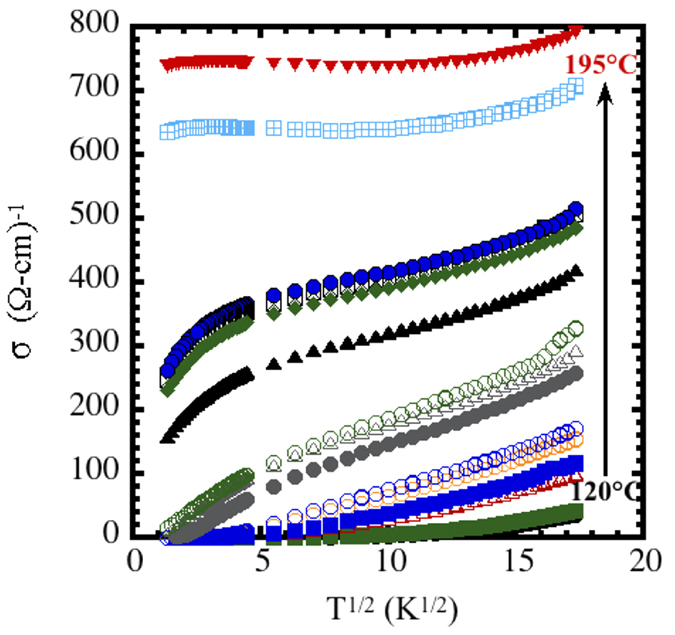
Plotting the conductivity data to the T^½^ behavior expected for three-dimensional systems near the MIT.

**Figure 5:**
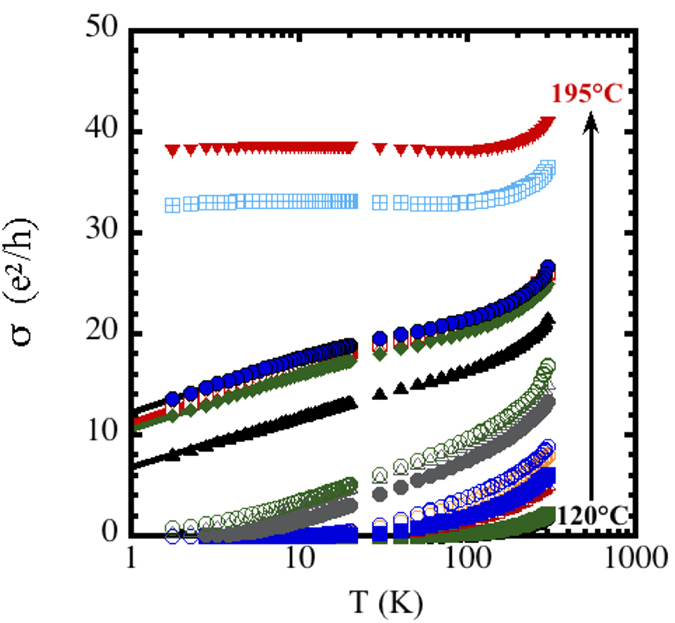
Plotting the conductivity data to the log(T) behavior expected for two-dimensional systems near the MIT. Conductivity per ◻ for the 20-nm thick RuO_2_ nanosheets plotted *vs*. log(T) as a function of calcination temperature. The samples were calcined from 120 °C to 195 °C in 5° steps (185 °C data are not included). The symbols are the same as in Fig. 4. The solid lines are extrapolated fits to σ = σ_0_ + σ_1_log(T) for T < 10 K.

**Figure 6:**
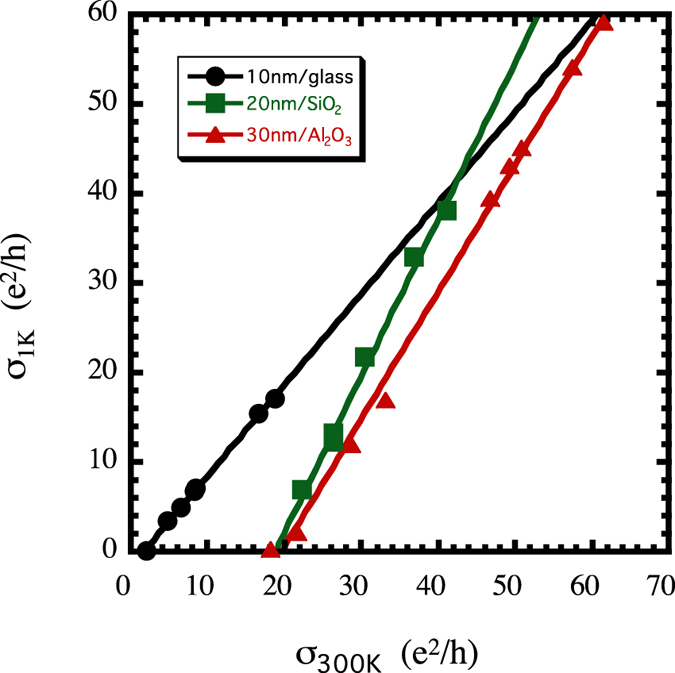
Continuity of the conductivity as disordered RuO_2_ nanosheets approach the MIT. Conductivity at 1 K vs. conductivity at 300 K exhibits the linear “mobility edge” observed in many three-dimensional systems. Note that the single-layer data are from several samples prepared together in the same deposition batch.

The sheet carrier concentration at 305 K, as determined from Hall measurements, increased from n ~ 10^14^–10^16^ cm^−2^ for the insulating samples to 10^17^–10^18^ cm^−2^ for the most conductive ones (Fig. 2b). Most of the calcinations produced films with hole carriers but several resulted in electron carriers. This variability in the dominant charge carrier is consistent with earlier work on thin-film RuO_2_ and is attributed to oxygen defects^36^,^37^. These values of carrier concentration are orders of magnitude larger than those reported for HMFETs (~10^10^–10^12^ cm^−2^)^2^,^3^,^4^,^5^,^6^,^7^,^31^ with the metallic samples having bulk values comparable to conventional metals (n_3D_ ~ 10^23^ cm^−3^). In the HMFET systems, the critical carrier densities were reported to be ~10^10^–10^12^ cm^−2^ while we find n~10^16^ cm^−2^ for the transitions from strongly localized to weakly localized, log(T), behavior.

The calculated room-temperature mobility of the carriers in RuO_2_ nanoskins as a function of calcination temperature shows significant scatter between ~0.01–10 cm^2^/(V-s) with the most metallic samples (those calcined at the highest temperature) exhibiting mobility between ~0.1 and 0.01 cm^2^/(V-s) (Fig. 2c). While these values are comparable to some reported for interfacial oxides^38^,^39^,^40^,^41^, they are many orders of magnitude smaller than those reported for HMFET devices, ~10^4^ cm^2^/(V-s)^2^,^3^,^4^,^6^,^7^,^31^. The values are also orders of magnitude smaller than those recently reported in gated structures for single- and double-layer MoS_2_, which exhibits an MIT with carrier concentrations on the order of 10^13^ cm^−2^ and mobilities that are in the range of 1–1000 cm^2^/(V-s)^42^,^43^,^44^,^45^. Although the carriers in the RuO_2_ nanoskins are present at high concentrations, approaching those of metals, their low mobility does not track that expected of metals, again highlighting the high disorder in the nanoscale oxide derived from the low-temperature solution-deposition protocol.

The quantity r_s_ = E_e−e_/E_F_ , where E_e−e_ is the characteristic electron–electron interaction energy and E_F_ is the Fermi energy, has been used to characterize transport in 2D systems^2^,^3^,^4^,^5^,^6^,^7^,^46^,^47^,^48^. While this quantity does not take into account disorder, the bare (high temperature), unrenormalized, value is useful for comparison with the other systems that have been studied. The parameter can be expressed as:


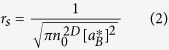


where 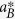 is the Bohr radius, 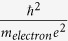, and 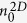 is the sheet carrier concentration^48^. We find r_305K_ ~ 1–10 for the insulating samples and 0.1–0.3 for the most conductive ones (Fig. 2d). The transition from strongly localized to weakly localized, log(T), behavior occurs for r_305K_ ~ 1 for both the 20- and 30-nm–thick RuO_2_ nanoskins. The transition from weakly localized to metallic behavior occurs for r_305K_ ~ 0.2 for the 20-nm–thick film and 0.5 for the 30-nm–thick film; in contrast the interfacial oxides have values of ~5–35 at the MIT^2^,^4^. It is not surprising that the critical values of r_s_ for the two situations are so different in that the levels of disorder are so different.

The strong deviation of the values of the bare carrier concentration, mobility, and r_s_ of these RuO_2_ films from those of the previously studied 2D systems leads us to conclude that disordered RuO_2_ exhibits a 2D MIT that falls in an as yet, unexplored region of transport phase space. The nature of the 2D MIT in the ballistic case has still not been definitely determined (*i.e.*, whether it is a “true” quantum phase transition or due to “conventional disorder”)^49^,^50^. Regardless of the microscopic details of the MIT, combining the HMFET results with those for the highly diffusive case presented here, it becomes clear that the 2D MIT is ubiquitous in the entire range of disorder.

## 2D phase diagram analogous to 3D

Several scaling approaches have been developed to describe transport in disordered 2D conductors^11^,^12^,^51^,^52^,^53^,^54^. The two phenomena modeled in these theories, weak localization and enhanced electron–electron interactions, are predicated on the presence of strong disorder, *i.e.*, highly diffusive transport, and are thus distinct from those developed for the HMFET results, which are in the ballistic limit. They all predict insulating behavior in two dimensions and are therefore, inadequate to describe the results reported here. Our results clearly show that the phase diagram of the 2D MIT for highly disordered, high carrier concentration materials is analogous to that for the 3D case shown in Fig. 1 with the amorphous metal phase replaced with an amorphous insulator phase that is characterized by the conductivity having a log(T) dependence. These results are consistent with more recent theory showing that a 2D MIT is possible, although it would need to be modified to apply to the highly disordered case^10^.

In summary, we have provided conclusive evidence for a continuous 2D metal–insulator transition in a low mobility, high carrier concentration material, highly disordered RuO_2_ nanoskins, findings that contradict the seminal work by Abrahams *et al.*^1^ The 2D metallic behavior occurs in a regime where mobility is orders of magnitude lower and carrier concentration is orders of magnitude larger than those observed in the previous systems that expressed 2D MITs, thus expanding the range of observed 2D metallic behavior. Our findings support more recent scaling arguments that predict a metallic state in 2D systems, and are key to understanding the transport properties of low-dimensional systems such as interfacial oxides.

## Additional Information

**How to cite this article**: Osofsky, M. S. *et al.* Disordered RuO_2_ exhibits two dimensional, low-mobility transport and a metal-insulator transition. *Sci. Rep.* **6**, 21836; doi: 10.1038/srep21836 (2016).

## Supplementary Material

Supplementary Information
