# Supplementary material for: Disordered RuO2 exhibits two dimensional, low-mobility transport and a metal–insulator transition
Source: Sci Rep. 2016 Feb 26;6:21836. doi: 10.1038/srep21836 (PMC4768250; doi:10.1038/srep21836)
Supplement: Supplementary Information [file srep21836-s1.pdf]

Supplemental Information
Disordered RuO_2 exhibits two dimensional, low-mobility transport and a metal-insulator transition

M. S. Osofsky,[1,*] C. M. Krowne,[2] K. M. Charipar,[1] K. Bussmann,[1] C. N. Chervin,[3] I. R. Pala,[3] and D. R. Rolison[3]

[1]Materials and Sensors Branch (Code 6360), [2]Electromagnetics Technology Branch (Code 6850), [3]Surface Chemistry Branch (Code 6170)
U. S. Naval Research Laboratory
Washington, DC 20375, USA

Estimate of sample dimensionality

Electron transport in highly disordered systems is described as a quantum diffusion problem. That is, electrons strongly scatter elastically from the disordered lattice in a phase coherent manner until a phase breaking (i.e., inelastic) event occurs. Thus, electrons coherently diffuse a distance called the Thouless length,

$$L_{Th} = \sqrt[2]{D\tau_{\varphi}} \tag{1}$$

where

$$D = \frac{1}{3} v_F^2 \tau_{el} \tag{2}$$

is the diffusivity, τ_{φ} is the phase coherence time, v_F is the Fermi velocity, and τ_{el} is the elastic scattering time.[1] If the sample thickness is larger than L_{Th} then it is three-dimensional and if it is less, then it is two-dimensional. Thus, by estimating L_{Th} we can determine the dimensionality of the RuO_2 films. The estimate is based on free electron parameters which will result in a lower limit on L_{Th}.[2]

Using the usual expression for v_F in an isotropic 3D system,

$$v_F = \frac{\hbar}{m} \sqrt[3]{3\pi^2 n} \tag{3}$$

where n is the carrier concentration and m is the electron mass. Combining equations 1-3 results in

$$L_{Th} = \sqrt[2]{\frac{\hbar^2}{3m^2} (3\pi^2 n)^{2/3} \tau_{el} \tau_{\varphi}}. \tag{4}$$

Since metallic behavior is not expected for disordered 2D systems, we shall examine the 20 nm film calcined at 190°C with $\sigma \sim 800\ (\Omega\text{-cm})^{-1}$ and $n \sim 10^{23}\ \text{cm}^{-3}$ that exhibits metallic behavior to confirm that can be treated as a 2D electronic system. The elastic and phase relaxation times cannot be directly determined from this work. Therefore, the values must be estimated from previous work on similar disordered systems. For instance, Kawaguti and Fujimori obtained $\tau_{el} \sim 10^{-13}$ s and $\tau_{\varphi} \sim 10^{-12}$ to 10^{-11} s for thin Ag and Au films below 10K from magneto-transport measurements.[3] The values of τ_{φ} are consistent with those found in other systems ($\sim 10^{-13}$ to 10^{-11} s).[4,5,6] Using these values gives $L_{Th} \sim 300$ nm-3000 nm which are significantly larger than the 20-30 nm thicknesses of the films used in this study.

Structural characterization

A Rigaku SmartLab X-ray diffractometer with fixed Cu$K\alpha$ radiation (λ = 154.06 pm) was employed for sample characterization. The scans were performed in Grazing Angle geometry in parallel-beam mode. The instrument was operated in a continuous mode in increments of 0.02 2θ, and counts were accumulated for 1 s at each step. The angle of incidence was set to different values (0.5°–1°) in order to vary the interaction length in each depth region with scanning of the exit angle.

Transport data obtained from 10nm and 30nm thick samples confirms the results reported for the 20nm samples (Figs. S2-S4). The data for the 10-nm data RuO_2 films are derived from three nanosheet samples (#8, #10, and #14) and two sputtered films.

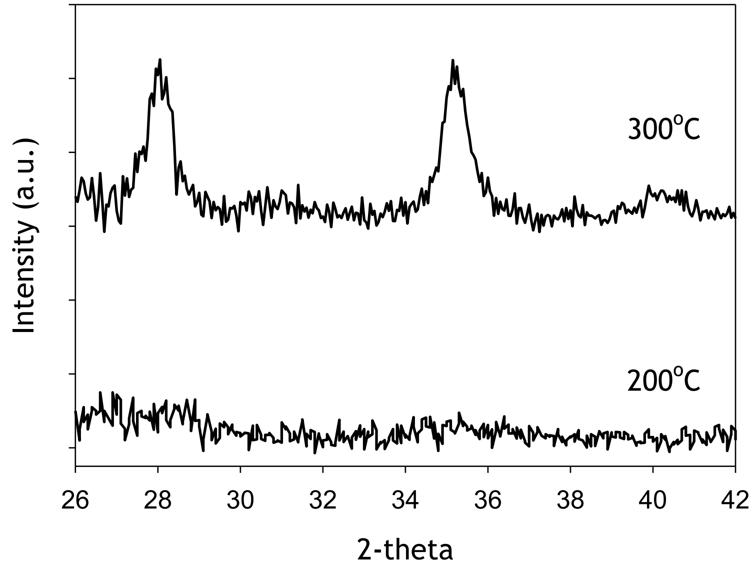

Figure S1 | Grazing-incident X-ray diffraction patterns for ~30-nm solution-deposited RuO_2 nanosheets deposited on SiO_2 substrates and calcined at (bottom) 200°C and (top) 300°C. The diffraction peaks for the 300°C-calcined RuO_2 nanosheet correspond to reflections for rutile RuO_2 (ICDD card # 00-043-1027) and are consistent with diffraction patterns for solution-deposited RuO_2 on SiO_2 fiber papers that were heated above 250°C (reference 27 in manuscript).

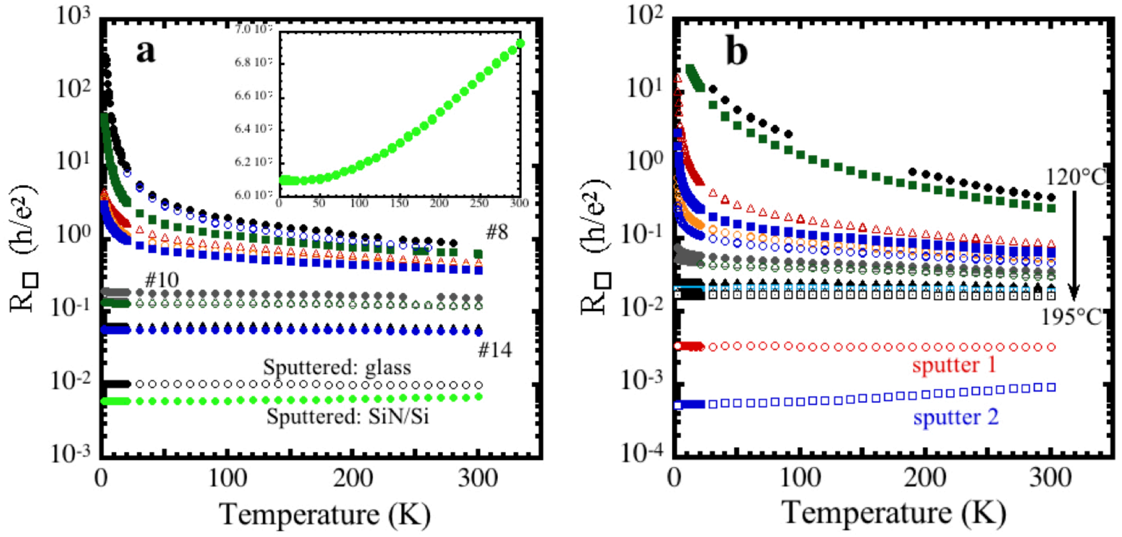

Figure S2 | Sheet resistance as a function of temperature of thin-film disordered and crystalline RuO_2. R/☐ (plotted in units of quantum resistance, h/e^2) obtained from 1.75K to305K for **a**, 10-nm and **b**, 30-nm solution-deposited RuO_2 nanosheets as a function of calcination temperature. Inset **a**: blow-up of the data for 10-nm sputtered RuO_2 film on SiN/Si substrate grown at 600°C; this film exhibits conventional metallic behavior with resistance decreasing with decreasing temperature.

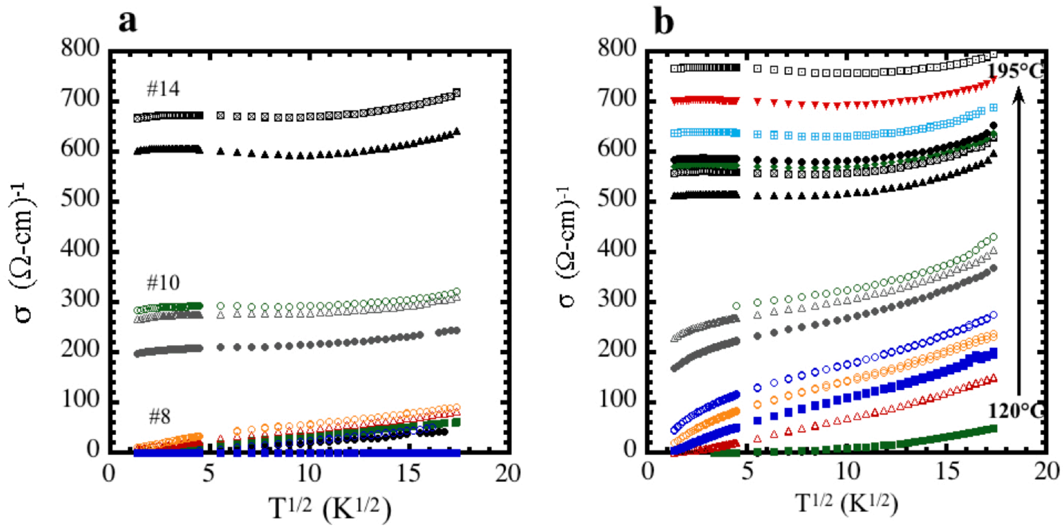

Figure S3 | Plotting the conductivity data to the T½ formalism expected for three-dimensional systems near the MIT. a, 10-nm and **b**, 30-nm thick RuO_2 nanosheets.

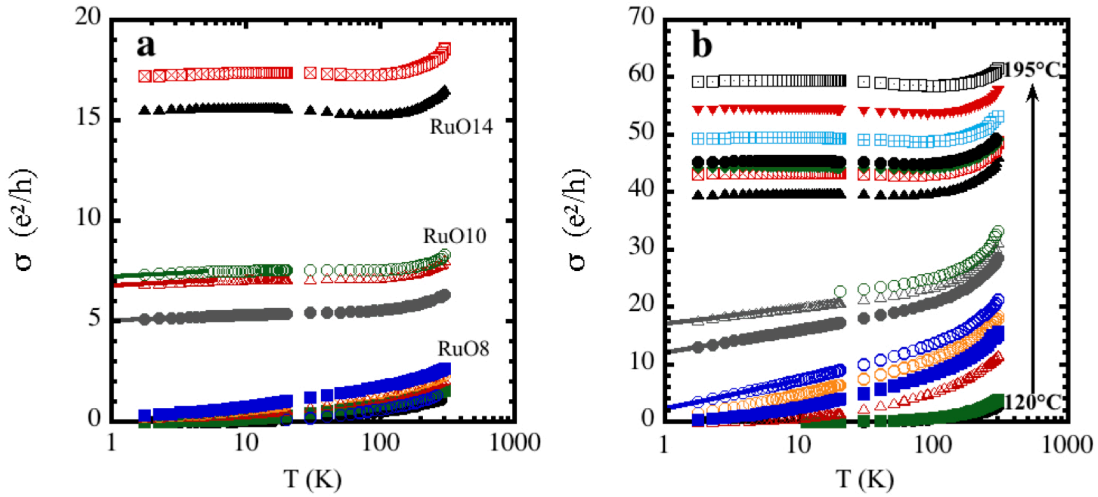

Figure S4 | Plotting the conductivity data to the log(T) formalism expected for two-dimensional systems near the MIT. Conductivity per ☐ for the **a**, 10-nm and **b**, 30-nm thick RuO_2 nanosheets plotted *vs*. log(T) as a function of calcination temperature. The 30nm samples were calcined from 120 °C to 195 °C in 5° steps. The solid lines are extrapolated fits to $\sigma = \sigma_0 + \sigma_1 \log(T)$ for $T < 10K$.

1. Lee, P.A. and Ramakrishnan, T.V. Disordered electronic systems. *Rev. Mod Phys.* **57**, 287–337 (1985).
2. Tardy, H. L., Measurements of magnetoresistance in amorphous metals, PhD thesis, University of Illinois, 1985.
3. Kawaguti, T. and Fujimori, Y., Magnetoresistance and inelastic scattering time in thin films of silver and gold in weakly localized regime, J. of Phys. Soc. Japan **52**, 722-725 (1983).
4. Osofsky, M., Tardy, H., LaMadrid, M., and Mochel, J. M., Strong and weak spin-orbit scattering near the metal-insulator transition, Phys. Rev. B **31**, 4715-4717 (1985).
5. Lin, J. J. and Giordano, N., Electron scattering times from weak localization studies of Au-Pd films, Phys. Rev. B **35**, 1071-1075 (1987).
6. Bergmann, G., Weak localization in thin films a time-of-flight experiment with conduction electrons, Phys. Rep. **107**, 1-58 (1984).
